# *Mycobacterium abscessus* DosRS two-component system controls a species-specific regulon required for adaptation to hypoxia

**DOI:** 10.3389/fcimb.2023.1144210

**Published:** 2023-03-09

**Authors:** Breven S. Simcox, Brooke R. Tomlinson, Lindsey N. Shaw, Kyle H. Rohde

**Affiliations:** ^1^ Division of Immunology and Pathogenesis, Burnett School of Biomedical Sciences, College of Medicine, University of Central Florida, Orlando, FL, United States; ^2^ Department of Cell Biology, Microbiology and Molecular Biology, University of South Florida, Tampa, FL, United States

**Keywords:** nontuberculous mycobacteria (NTM), *Mycobacterium abcessus*, hypoxia, two-component system (TCS), RNAseq, DosR

## Abstract

*Mycobacterium abscessus* (*Mab*), an emerging opportunistic pathogen, predominantly infects individuals with underlying pulmonary diseases such as cystic fibrosis (CF). Current treatment outcomes for *Mab* infections are poor due to *Mab’s* inherent antibiotic resistance and unique host interactions that promote phenotypic tolerance and hinder drug access. The hypoxic, mucus-laden airways in the CF lung and antimicrobial phagosome within macrophages represent hostile niches *Mab* must overcome *via* alterations in gene expression for survival. Regulatory mechanisms important for the adaptation and long-term persistence of *Mab* within the host are poorly understood, warranting further genetic and transcriptomics study of this emerging pathogen. DosRS*_Mab_*, a two-component signaling system (TCS), is one proposed mechanism utilized to subvert host defenses and counteract environmental stress such as hypoxia. The homologous TCS of *Mycobacterium tuberculosis* (*Mtb*), DosRS*_Mtb_*, is known to induce a ~50 gene regulon in response to hypoxia, carbon monoxide (CO) and nitric oxide (NO) *in vitro* and *in vivo*. Previously, a small DosR*_Mab_* regulon was predicted using bioinformatics based on DosR*_Mtb_* motifs however, the role and regulon of DosRS*_Mab_* in *Mab* pathogenesis have yet to be characterized in depth. To address this knowledge gap, our lab generated a *Mab dosRS* knockout strain (*Mab_ΔdosRS_)* to investigate differential gene expression, and phenotype in an *in vitro* hypoxia model of dormancy. qRT-PCR and lux reporter assays demonstrate *Mab_dosR* and 6 predicted downstream genes are induced in hypoxia. In addition, RNAseq revealed induction of a much larger hypoxia response comprised of >1000 genes, including 127 differentially expressed genes in a *dosRS* mutant strain. Deletion of DosRS*_Mab_* led to attenuated growth under low oxygen conditions, a shift in morphotype from smooth to rough, and down-regulation of 216 genes. This study provides the first look at the global transcriptomic response of *Mab* to low oxygen conditions encountered in the airways of CF patients and within macrophage phagosomes. Our data also demonstrate the importance of DosRS*_Mab_* for adaptation of *Mab* to hypoxia, highlighting a distinct regulon (compared to *Mtb)* that is significantly larger than previously described, including both genes conserved across mycobacteria as well as *Mab*-specific genes.

## Introduction

*Mycobacterium abscessus* (*Mab*) is an opportunistic pathogen capable of causing skin, soft tissue and pulmonary infections in immunocompromised individuals and individuals with pre-existing lung disease such as cystic fibrosis (CF) and bronchiectasis (Brown-Elliott and Wallace, 2002; Olivier et al., 2003; Harris and Kenna, 2014; Lee et al., 2015; Lopeman et al., 2019). Impaired innate immune defenses and viscous mucus within the CF lung contribute to reduced clearance of bacterial pathogens leading to increased rates of infections and morbidity (Lyczak et al., 2002; Chmiel and Davis, 2003). *Mab* is the most common rapidly-growing mycobacterial (RGM) species recovered from the lungs of CF patients (Esther et al., 2010). Within the CF population and patients with underlying lung dysfunction, infections caused by *Mab* are associated with lung function decline, increased hospital visits, prolonged hospital stays and in some cases exclusion from lung transplants (Olivier et al., 2003; Esther et al., 2010; Lopeman et al., 2019). Due to *Mab’s* inherent antibiotic resistance, treatment options are limited, resulting in extremely low cure rates of less than 50% (Greendyke and Byrd, 2008; Hurst-Hess et al., 2017; Molina-Torres et al., 2018; Story-Roller et al., 2018; Lopeman et al., 2019).

The development of effective treatment strategies for *Mab* is hindered by discrepancies between *in vitro* and *in vivo* susceptibilities associated with *Mab’s* unique lifestyle (Greendyke and Byrd, 2008; Philley et al., 2016; Molina-Torres et al., 2018). Akin to *Mycobacterium tuberculosis* (*Mtb*), the causative agent of tuberculosis, *Mab* resides within pulmonary macrophages and within granulomas which limit antibiotic accessibility and promote drug tolerance, making treatment of an inherently antibiotic resistant pathogen even more difficult (Bernut et al., 2016; Peddireddy et al., 2017). The viscous mucus in the CF lung represents an additional hostile niche within the host to which *Mab* must adapt (Lyczak et al., 2002; Chmiel and Davis, 2003; Miranda-CasoLuengo et al., 2016; Dubois et al., 2019). One key host-derived stress encountered by *Mab* in all three of these microenvironments is decreased oxygen tension, with oxygen tension estimated to be ~1% O_2_ within these niches (Worlitzsch et al., 2002; Cunningham-Bussel et al., 2013; Hudock et al., 2017). Thus, to successfully cause an infection, *Mab* must encode mechanisms to adapt and persist under hypoxic conditions. Transcriptional responses to host-derived cues/stresses are not well-defined in this NTM pathogen, requiring further studies to understand how *Mab* adapts its physiology and virulence factor expression to cause insidious, persistent infections.

Two-component signaling (TCS) is a mechanism commonly utilized by prokaryotes to regulate virulence gene expression in response to host-derived cues (Yarwood et al., 2001; Walters et al., 2006; Gonzalo-Asensio et al., 2008; Gooderham and Hancock, 2009). A typical TCS consists of a sensor histidine kinase (HK) responsible for signal recognition and subsequent phosphorylation of a cognate response regulator (RR) which binds DNA motifs within promoter regions to drive alterations in gene expression (Stock et al., 1989; West and Stock, 2001; Mascher et al., 2006; Salazar and Laub, 2015). *Mab* encodes 11 TCS, 5 orphan RRs and 1 orphan HK each with a corresponding ortholog in *Mtb*; however, in-depth studies of *Mab* TCS have not been performed (Bretl et al., 2011). The well-documented atypical TCS DosRS/T*_Mtb_*, is known to control a ~50 gene regulon to counteract hypoxic and nitrosative stress encountered within macrophages and granulomas (Sherman et al., 2001; Park et al., 2003; Voskuil et al., 2003; Leistikow et al., 2010; Rohde et al., 2012; Cunningham-Bussel et al., 2013; Peterson et al., 2020; Kundu and Basu, 2021). DosRS/T*_Mtb,_* contains 2 HKs (DosS and DosT) rather than one which are responsible for phosphorylating the RR DosR at different stages of hypoxia (Roberts et al., 2004). Although *Mtb dosT* contributes to signaling in early stages of hypoxia, it is constitutively expressed and is not part of the DosR*_Mtb_* regulon (Honaker et al., 2009). The DosR*_Mtb_* regulon includes autoregulation of *dosRS* itself, as well as heat shock proteins, triacylglycerol synthases, ferrodoxins, universal stress proteins, diacylglycerol acyltransferases, and nitroreductase which are implicated in dormancy, resuscitation, phenotypic drug tolerance and increased lipid metabolism (Park et al., 2003; Voskuil et al., 2003; Leistikow et al., 2010; Galagan et al., 2013; Aguilar-Ayala et al., 2017). Induction of *Mtb dosR* within animal models capable of forming hypoxic granulomas (rhesus macaques, guinea pigs, and C3HeB/FeJ mice) and attenuation of mutants lacking DosRS*_Mtb_* highlight the importance of this TCS for *Mtb* pathogenesis (Converse et al., 2009; Gautam et al., 2015a, Gautam et al., 2015b; Mehra et al., 2015).

According to whole genome sequence data, *Mab* encodes a DosR ortholog (*Mab*_3891c) with a high level of homology with DosR*_Mtb_* (~72% identity) adjacent to and upstream of DosS*_Mab_* (*Mab*_3890c) with lower similarity (~51% identify) to its counterpart in *Mtb*. *Mab* does not appear to encode a secondary orphan HK analogous to DosT, with the closest homolog to *dosT* being *dosS_Mab_/Mab*_3890C (53% identity). At the time this study was initiated, only two reports made mention of DosRS*_Mab_*. A bioinformatics study by Gerasimova et al. used *Mtb* DosR promoter motifs to predict a small DosR*_Mab_* regulon consisting of only 6 genes (Gerasimova et al., 2011). A subsequent transcriptomics study by Miranda Caso-Luengo et al. demonstrated induction of the 6 predicted DosR*_Mab_* regulated genes plus 56 other genes upon exposure to nitric oxide (NO) (Miranda-CasoLuengo et al., 2016). It remained unclear whether induction of these genes occurs through signaling of DosRS*_Mab_* or whether hypoxia is an induction cue.

Although there is considerable overlap in the repertoires of TCS encoded by different mycobacteria, few cross-species transcriptomic studies are available comparing TCS regulons and regulatory networks between e.g. *Mtb* and NTM pathogens. Given the diversity of conditions encountered by *Mab* as an environmental, opportunistic pathogen, and larger genome (compared to *Mtb)* comprised of ~800 species-specific genes, the potential for unique gene sets in *Mab* TCS regulons is high (Malhotra et al., 2017; Wee et al., 2017). A hypoxia model mimicking the physiologic conditions in the mucus of CF airways, within granulomas and intramacrophage compartments was used to evaluate the role of DosRS*_Mab_* and assess transcriptional regulation mediated by this TCS. Our work demonstrates DosRS*_Mab_* is important for maximal growth and survival in hypoxia and regulates a potentially larger set of genes than previously predicted. RNAseq revealed upregulation of >1000 genes in hypoxia including 127 putative DosRS*_Mab_* regulated genes. Information gained from this study identifies the importance of the DosRS*_Mab_* TCS in adaptation to hypoxia for survival and provides valuable knowledge of a novel set of hypoxia-induced genes in this species for future studies.

## Methods

### *Mycobacterium abscessus* cloning

*Mab_ΔdosRS_* was generated *via* recombineering as described by van Kessell and Hatfull in the strain *Mab* 390S obtained from the Thomas Byrd lab (Byrd and Lyons, 1999; van Kessel and Hatfull, 2007). In brief, an allelic exchange substrate (AES) was engineered containing an apramycin resistance cassette flanked by ~1000 nucleotides upstream and downstream of the *dosRS* operon *via* round the horn PCR and fast cloning (Moore and Prevelige, 2002; Li et al., 2011). *Mab::*pJV53 competent cells induced with .02% acetamide for 4 hours were electroporated with 100ng AES, recovered in 7H9 OADC media for 24 hours, and plated on 7H10 agar supplemented with apramycin 50 µg/ml. Complement strain, *Mab_ΔdosRS+C_*, containing *dosRS* with its native promoter was engineered using round the horn (Moore and Prevelige, 2002) and fast cloning (Li et al., 2011) in the integrating vector pUAB400 followed by electroporation into *Mab_ΔdosRS_* (Singh et al., 2006). *Mab* 390S and *Mab_ΔdosRS_* were transformed with pMV306hspG13lux (Addgene #26161) to generate the constitutive lux strains, *Mab* 390S P_hsp60_-lux and *Mab_ΔdosRS_* P_hsp60_-lux. Lux reporters under the control of P_dosR_ and P_2489_ were constructed in the background plasmid pMV306hspG13lux to generate Mab 390S P_dosR_-lux, *Mab_ΔdosRS_* P_dosR_-lux, *Mab* 390S P_2489_-lux, and *Mab_ΔdosRS_* P_2489_-lux, *via* replacement of P_hsp60_ (hsp60 promoter) using round-the horn cloning (Moore and Prevelige, 2002; Andreu et al., 2010; Li et al., 2011). Refer to **Table S1** for strains, plasmids and primers used for cloning.

### Hypoxic and re-aeration culture models

Cultures were grown in 7H9-OADC+.05% tyloxapol from glycerol stocks at 37°C while shaking unless otherwise noted. For growth kinetics assays, strains were inoculated from mid-log phase starter cultures into 13ml of media in filter-capped T-25 flasks to an OD_600_ = 0.02. Hypoxic cultures were grown standing in a hypoxic incubator set at 1% O_2_ while aerated controls were cultured at 20% O_2_ while shaking at 100 rpm. Re-aeration studies were conducted after cultures were subjected to hypoxia or grown at 20% O_2_ for 30 days. Optical density readings (OD_600nm_) were taken on days 2, 3, 5, 8 and 10.

### RNA experiments

RNA was extracted as previously described (Rohde et al., 2007) in triplicate from hypoxic cultures (1% O_2_) and normoxic cultures (20% O_2_) for qRT-PCR and RNAseq. At designated time points, cultures were pelleted at 4300 rpm for 5 minutes, resuspended in guanidine thiocyanate buffer, pelleted again at 12,000 rpm for 5 min, and stored at -80°C until processing. Thawed pellets were resuspended in 65°C Trizol then lysed using 0.1mM silicon beads in a BeadBeater at max speed for 1 minute 2x followed by cooling on ice for 1 minute between bead beating. Isolation of total RNA from Trizol lysates was performed using chloroform extraction and Qiagen RNeasy column purification. Total RNA was treated with Turbo DNase (Invitrogen) to eliminate DNA contamination. 50 ng/µl of total RNA was used to generate cDNA using iScript™ cDNA synthesis kit (Bio-Rad) for qRT-PCR reactions carried out in a QuantStudio7 thermocycler. Primers used for qRT-PCR are listed in **Table S1**. RNA samples for RNAseq analysis were pooled at equal RNA concentrations from three biological experiments as previously described (Tomlinson et al., 2021; Tomlinson et al., 2022) prior to library preparation at a concentration of 50 ng/µl in 20 µl. Only RNA samples with RIN>6 as determined by Tapestation analysis were utilized. RNA samples were sequenced by Microbial Genome Sequencing Center (MiGs) using Illumina sequencing protocol aligning reads to the *Mab* ATCC19977 genome (accession #CU458896). RNAseq data reflects a minimum of 12M paired end reads per sample. Due to incompatibility of Ribo-zero rRNA removal kit (Illumina) with *Mab* which resulted in high levels of rRNA, MiGS designed custom depletion probes (**Table S2**) for rRNA depletion. Raw data was received from MiGs as fastq files followed by analysis using CLC Genomics Workbench 12 (Qiagen Bioinformatics). Illumina paired importer tool was used to eliminate failed reads using the quality score parameter option set to Illumina Pipelines 1.8. Expression browser tool (v1.1) was used to calculate gene expression with an output of transcript per million (TPM). Differential gene expression is expressed as a log_2_FC of ≤-1 or ≥1 and visualized as scatter plots created in GraphPad Prism 9.

### Lux reporter assays

Bio-luminescent reporter strains were grown to mid-log phase, diluted to .02 OD in 13ml in T25 flasks and grown in either 20% O_2_ or 1% O_2_ for 1, 5 and 20 days. 200 ul of each culture was aliquoted in triplicate into 96 well white bottomed plate to measure luminescence *via* Synergy H4 reader (Biotek). Fold change of luminescence was analyzed comparing individual strains in 1% O_2_ to 20% O_2_ using *Mab* 390S P_hsp60_-lux or *Mab_ΔdosRS_* P_hsp60_-lux as internal controls (1%O_2_(P*_dosR_* or P*_2489_*/P*_hsp60_*))/(20% O_2_(P*_dosR_* or P*_2489_*/P*_hsp60_*)).

## Results

### Growth and transcriptome remodeling of *Mab* in a defined hypoxia model

Our first goal was to develop a tractable *in vitro* model to investigate the mechanisms employed by *Mab* to persist under physiologically relevant oxygen-limited conditions. To do this, *Mab* 390S was grown under standing conditions in a 1% O_2_ atmosphere to mimic the pO_2_ observed within the CF lung, macrophages and granulomas (Worlitzsch et al., 2002; Cunningham-Bussel et al., 2013; Hudock et al., 2017). *Mab* 390S grew steadily at 1% O_2_, reaching OD_600_ ~0.7 by day 5 with continued increase to ~0.9 by day 10 (**Figure S1**). Somewhat unexpectedly, the intended aerobic control culture (20% O_2_ atmosphere, standing) had a very similar growth profile, reaching only a slightly higher OD ~1.2 by day 10 (**Figure S1**). In contrast, *Mab* 390S grown in 20% O_2_ with shaking (100rpm) reached a maximum density by day 5 (OD_600_~1.6) and subsequently plateaued up to day 10 (**Figure S1**). This difference is likely due to the microaerobic conditions experienced by bacilli growing below the media surface, as previously seen with *Mtb* and BCG strains grown in standing conditions (Cunningham and Spreadbury, 1998; Purkayastha et al., 2002). To maximize the contrast between aerobic and hypoxic conditions, *Mab* cultures shaking in 20% O_2_ served as references for all hypoxia experiments. Thus, *Mab* is not only able to survive but to actively replicate under *in-vivo* like hypoxia conditions. It is worth noting that exposure of *Mab* to sudden hypoxia using BD Gaspack anaerobic pouches led to rapid sterilization of the cultures (data not shown). This may indicate a lower threshold of O_2_ levels needed for *Mab* viability was exceeded or that slower, adaptive responses are necessary to survive.

**Figure 1:**
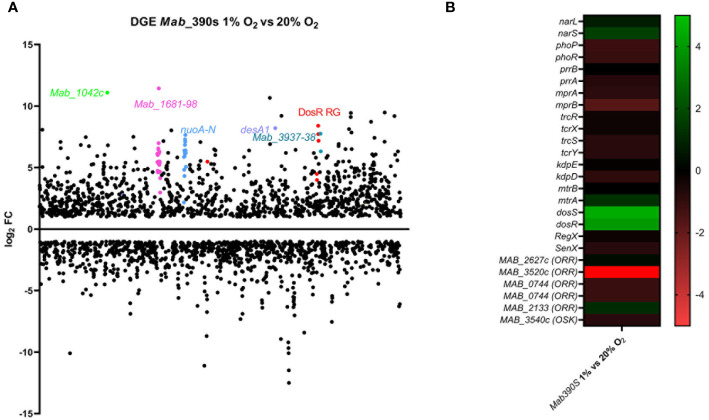
Transcriptome analysis of *Mab*390S in defined hypoxia model. DGE is visualized as **(A)** scatter plot depicting changes in gene expression reported as TPM with ≥ or ≤ log_2_FC on Day 5 for *Mab* 390S 1% O_2_ vs *Mab* 390S 20% O_2._ Dots represent individual genes (neon green=*Mab*_*1042C*, pink=*Mab_1681-1698*, blue=*nuo* operon, purple= *desA1*, turquoise=*Mab_3937* & *3938*, red= predicted DosR regulated genes (RG). **(B)** Heat map showing DGE of TCS of *Mab* 390S in 1% O_2_ vs 20% O_2_. In gene names, orphan response regulators and orphan sensor kinases are denoted as ORR and OSK, respectively.

Exploiting this model to profile *Mab* differential gene expression (DGE) in response to hypoxia, RNAseq transcriptomic analysis was conducted on wild-type *Mab* 390S cultured at 1% O_2_ and 20% O_2_ on day 5. This time point reflects the maximal difference in OD_600nm_ between hypoxic and aerated cultures and precedes the plateau in growth curves (**Figure S1**). The scatter plot in **Figure 1A** graphically depicts the dramatic genome-wide alterations in *Mab* 390S gene expression induced by *in vivo-*like low oxygen condition. The *Mab* hypoxia response included induction of 1,190 genes (≥ 1 log_2_ fold change compared to 20% O_2_) and downregulation of 1,062 genes (≤ 1 log_2_ fold change compared to 20% O_2_). This represented a larger set of hypoxia-induced genes than observed in *Mtb* in two studies under similar 1% O_2_ hypoxia conditions (induction of ~400 genes detected *via* microarray and ~682 *via* RNAseq) (Rustad et al., 2009; Vilcheze et al., 2022). These data highlight the functional genomic differences between *Mab* and *Mtb*, in particular their distinct patterns of gene regulation in response to hypoxia which are discussed in detail below.

To identify putative regulators of hypoxia adaptation in *Mab*, we assessed the differential expression of TCSs and other annotated transcription factors in the 1% hypoxia model. Of the 11 TCS orthologous to *Mtb*, only *dosRS*, *mtrA*, *narS* and the orphan RRs *Mab*_*2133* and *Mab*_*3520c* displayed DGE (**Figure 1B**). *Mab*_*dosRS* exhibited the largest magnitude of gene induction with log_2_FC of 3.9 and 4.5, respectively, pointing to DosRS*_Mab_* as an important TCS facilitating adaptation to hypoxic stress. The other TCS components were minimally induced with a log_2_FC of 1.6 for *narS*, 1.2 for *mtrA* and 1.1 for the orphan RR *Mab*_*2133*. The roles of *narS, mtrA* and *Mab*_*2133* have not been determined in *Mab*. However, in the context of *Mtb*, the regulons of DosRS and NarLS TCS display partial overlap and protein-protein interactions between the RR from these two TCSs, DosR and NarL, have been detected (Malhotra et al., 2015). *mtrA* is essential in *Mtb* due its role in replication, whereas in *Mab mtrA* was reported to be non-essential, pointing to potential differences in the role of this TCS between the two species (Fol et al., 2006; Akusobi et al., 2022). The *Mtb* ortholog (Rv3143) of orphan RR *Mab*_*2133* is implicated in nitrate metabolism in the absence of oxygen, binds to *nuo* subunits required for electron transport and is within nuo operon (Plocinska et al., 2022). *Mab’s* nuo operon displays synteny with the *Mtb nuo* operon including the orphan RR *Mab*_*2133*. Whereas Rv3143 was moderately induced by hypoxia in a DosR-dependent manner (Kendall et al., 2004), the upregulation of *Mab_2133* in our hypoxia model was not altered in the absence of DosRS (**Table S3**). The only TCS gene displaying substantial downregulation was *Mab*_*3520c* (log_2_FC = -9.7). The *Mtb* ortholog of *Mab*_*3520c*, Rv0260c, is known to be upregulated in hypoxia and to interact with DosS *via* protein-protein interaction independent of DosR; however, the function of *Rv0260c* has not been identified (Gautam et al., 2019; Vilcheze et al., 2022). The opposite pattern of regulation of Rv0260c (induced) and *Mab*_3520c (repressed) in hypoxia implies they are utilized differently for adaptation to hypoxia. Thus, our transcriptomic analyses of *Mab* under physiologic hypoxia conditions point to *Mab* DosRS as an important TCS aiding in adaptation to hypoxia.

In addition to induction of the *Mab*_*dosRS* TCS, we also observed upregulation of 80 single component transcription factors (TF) in hypoxia (**Table S3**). Due to the high number of TF, only the genes with a log_2_FC ≥ 3 are included in **Table S4** with the most highly induced genes being *Mab*_*4180* (lclR), *Mab*_*2606c* (TetR family), *Mab_4332* (TetR family) and *Mab*_*3018* (GntR family). The *Mtb* orthologs for *Mab*_*4332* (Rv0273c) and *Mab*_*3018* (Rv0586) have 64.14% and 45.53% identity, respectively. Rv0273c has been identified as a regulator of *inhA*, an enoyl-ACP reductase involved in mycolic acid synthesis, and Rv0586 is known to mediate lipid metabolism in *Mtb* (Santangelo Mde et al., 2009; Yousuf et al., 2018; Zhu et al., 2018). Both *Mab*_*4180* and *Mab*_*2606c* share less than 30% sequence homology with the *Mtb* orthologs and no known function has been identified. Although few *Mab* transcriptional regulators have been characterized, the large number of regulators with altered expression under hypoxic stress are likely key nodes in the regulatory networks needed to adapt *in vivo*.

Due to the number of TFs and their magnitude of modulation in response to hypoxia, including upregulation of *Mab*_*dosRS*, the broad scope of transcriptional changes was not surprising. The list of hypoxia-induced genes included loci involved in fatty acid and cholesterol metabolism, components of the NADH-quinone oxidoreductase subunits (*nuoA*-*N*), 6 epoxide hydrolases (*ephD*), ATP synthase subunits, 5 mammalian cell entry operons (MCE), members of the glycopeptidolipid locus (GPL), and 520 hypothetical genes (**Tables S3, 4**). Several pathways critical for pathogenesis of *Mtb*, such as fatty acid and cholesterol metabolism, are also induced in various hypoxia models (Wayne model, 1% O_2_) and within granulomas (Wilburn et al., 2018; Yang et al., 2021; Vilcheze et al., 2022). All four MCE loci in *Mtb* are differentially expressed in response to hypoxia, suggesting an important role for this family of lipid/cholesterol transporters in adaptation to this stress. While *mce2* and *mce3* were induced in a hypoxia model similar to ours (1% O_2,_ 5 days), *mce1* was repressed, and *mce4* expression remained unchanged (Vilcheze et al., 2022). A separate study by Rathor et al. found that *mce4* was upregulated after much longer durations of hypoxia stress (Rathor et al., 2016). Mce1 and Mce4 are known to play a role in the transport of fatty acids and cholesterol (Wilburn et al., 2018; Klepp et al., 2022), whereas the function of Mce2 and Mce3 have not been determined yet. In contrast, five of the seven MCE systems encoded by *Mab* were upregulated in 1% O_2_ after 5 days (**Tables S3, 4**). Although the biological role of MCE complexes in *Mab* have not been studied, their distinct expression profile suggest they may be important for *in vivo* survival. The *Mab* hypoxia-induced gene set also included a large number of genes implicated in β-oxidation pathways - 14 *fadE* genes, 8 *fadD* genes, and one of each *fadA* and *fadH* (**Tables S3, 4**). Upregulation of 6 *ephD* genes (an epoxide hydrolase predicted to alter the amount of epoxymycolates in the cell wall), members of the GPL locus accounting for smooth morphology (**Tables S3, 4**), and arabinosyltransferases A and B (**Supplemental Table 1**) involved with arabinogalactan synthesis indicate *Mab* may undergo cell wall remodeling in response to hypoxia (Amin et al., 2008; Gutierrez et al., 2018; Madacki et al., 2018).

In addition to β-oxidation, metabolic pathway induction included *nuo* subunits *A*-*N*, ATPase subunits, and cytochrome P450 genes (**Tables S3, 4**). NuoA-N are subunits of the proton pumping NADH dehydrogenase type 1 responsible for transferring electrons to menaquinone in the electron transport chain (ETC) in an energy conserving manner to generate a PMF (Weinstein et al., 2005). Although these genes are upregulated in the RGM *Mycobacterium smegmatis* (*Msmeg)* during slowed growth and in *E.coli* in anaerobic conditions, this is not a feature observed in the hypoxic response of slow-growing mycobacteria (SGM) like *Mtb* (Unden and Bongaerts, 1997; Berney and Cook, 2010). In contrast to *Mab*, the nuo operon and ATPase subunits are downregulated in hypoxic *Mtb*, further highlighting the distinct stress responses and energy metabolism of these two species in response to hypoxia (Cook et al., 2014; Vilcheze et al., 2022). Of the 25 *Mab* cytochrome P450s, 14 were induced in hypoxia (**Supplemental Table 1**). This data is consistent with hypoxic induction a large number of cytochrome P450s in the RGM *Msmeg* but not in the SGM *Mtb* with the exception of only 2 cytochrome P450s (Sherman et al., 2001; Berney and Cook, 2010; Ortega Ugalde et al., 2019). The roles of individual *Mab* cytochrome P450s remain unknown but the functions of the *Mtb* orthologs are dependent on their ferredoxin redox partners and include cholesterol degradation, redox balance, and virulence (Capyk et al., 2009). Our data supports hypoxic induction of 2 ferredoxins (*Mab*_*0914c* & *Mab*_*2049c*) and 3 ferredoxin reductases (*Mab*_*0930*, *Mab*_*2047c* and *Mab*_*4356c*) (**Table S3**). Induction of *nuo*A-N, ATPAse subunits, the large number of cytochrome P450s and ferredoxins implies *Mab* may employ different sets of genes for anaerobic respiration in its response to hypoxia and adapation.

*Mab’s* transcriptional adaptation to hypoxia also comprised a large set of downregulated genes including but not limited to multiple TF, tRNAs, 30S and 50S ribosomal proteins, alternative sigma factors, and 431 hypothetical proteins (**Table S3**). Downregulation of genes involved in essential processes such as protein synthesis (e.g. tRNAs, ribosomal proteins and sigma factors) are consistent with the slowed growth observed in hypoxic *Mab* cultures. Included among the most downregulated genes in hypoxia (**Table S3**) is the orphan response regulator *Mab_3520c* (**Figure 1B**) and three adjacent upstream genes (nirD/MAB_3521c, nirB/MAB_3522c), and nark3/MAB_3523c) predicted to be involved in nitrite reduction and extrusion (Malm et al., 2009). *Mtb nirB* and *nirD* orthologs are reportedly induced in nutrient starvation but minimal to no DGE occurred in hypoxia at 1% O_2_ (Vilcheze et al., 2022). However, in the Wayne model of hypoxia *Mtb nirB* displayed induction and functional *nirBD* genes were required for growth in hypoxia when nitrite was used as the sole nitrogen source (Akhtar et al., 2013).

### Construction and validation of *Mab*_ΔdosRS_ and *Mab*_ΔdosRS+C_

**Figure 2:**
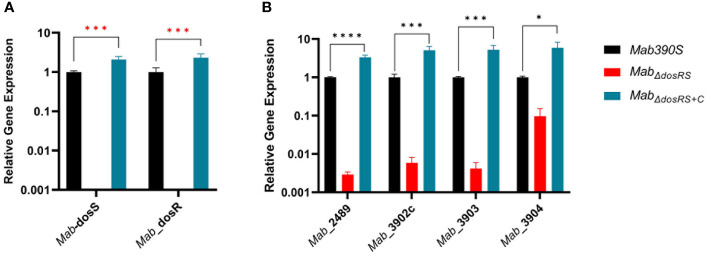
Validation of *Mab_ΔdosRS_* and *Mab_ΔdosRS+C_*. qRT-PCR was performed to **(A)** confirm the deletion and restoration of *dosS* and *dosR* in *Mab*_ΔdosRS_ and *Mab*_ΔdosRS+C_, respectively, and **(B)** assess the effect on predicted downstream genes. *Mab 390S* (black)*, Mab_ΔdosRS_* (red) and *Mab_ΔdosRS+C_* (blue). Data is representative of 3 experiments performed in triplicate. *P* values were calculated *via* one -way ANOVA using GraphPad. Red stars indicate C_t_ values were not detected for *Mab dosR* nor *dosS* in the mutant strain. *P-value <.05, ***P-value <.001 ****P-value <.0001.

Elucidation of the global transcriptional responses of *Mab* to hypoxia for the first time revealed the DosRS TCS is employed during hypoxic adaptation, yet much remains unknown about the role of DosRS in gene regulation and *Mab* pathogenesis. The DosR*_Mab_* regulon was previously predicted to consist of only 6 genes - *Mab*_3890 (*dosS*), *Mab*_3891 (*dosR*), *Mab*_2489 (universal stress protein, USP), *Mab*_3902c (ortholog of Rv2004c), *Mab*_3903 (nitroreductase) and *Mab*_3904 (USP) – based solely on bioinformatic analysis (Gerasimova et al., 2011). However, at the time this study was initiated, the role of DosRS*_Mab_* signaling in gene regulation, including induction of this gene set, remained to be experimentally demonstrated. To enable determination of the DosRS*_Mab_* regulon and role of this TCS in *Mab* adaptation to hypoxia, we generated *Mab_ΔdosRS_* (knockout mutant) using recombineering and the corresponding complemented strain (*Mab_ΔdosRS_*_+C_) expressing a single, integrated *dosRS* allele driven by its native promoter (van Kessel and Hatfull, 2007). In addition to confirming strain genotypes by PCR and DNA sequencing (data not shown), the absence of *dosRS* transcripts in *Mab_ΔdosRS_* and restoration to wild-type levels in *Mab_ΔdosRS_*_+C_ was validated by qRT-PCR (**Figure 2A**). We next assessed transcript levels of 4 genes (in addition to *dosRS* operon itself) previously predicted to be DosR-dependent. Loss of a functional DosRS system resulted in down-regulation of all predicted DosR*_Mab_*-regulated genes by >2 log (*MAB_2489, MAB_3902c, MAB_3903)* or > 1 log in the case of *MAB_3904* with restoration to wild-type levels in the complemented strain (**Figure 2B**), consistent with DosR-mediated induction of these genes.

### DosRS*_Mab_* is required for maximal growth in hypoxia

**Figure 3:**
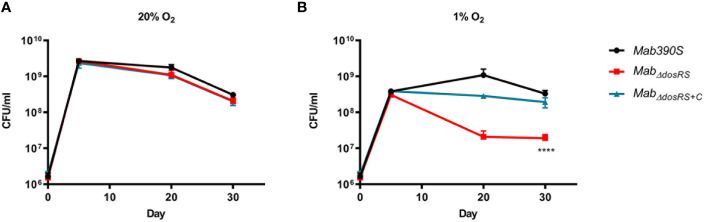
*Mab_ΔdosRS_* is attenuated in hypoxia. Growth kinetics in hypoxia were assessed *via* serial dilutions, spot plating and enumeration of CFU/ml on day 5, 20 and 30. **(A)** Growth kinetics at 20% O_2_ **(B)** Growth kinetics at 1% O_2._ *Mab 390S* (black)*, Mab_ΔdosRS_* (red) and *Mab_ΔdosRS+C_* (blue). Data reflects 3 independent experiments performed in triplicate. *P-*values were calculated *via* one-way ANOVA using GraphPad, ****P-value <.0001.

**Figure 4:**
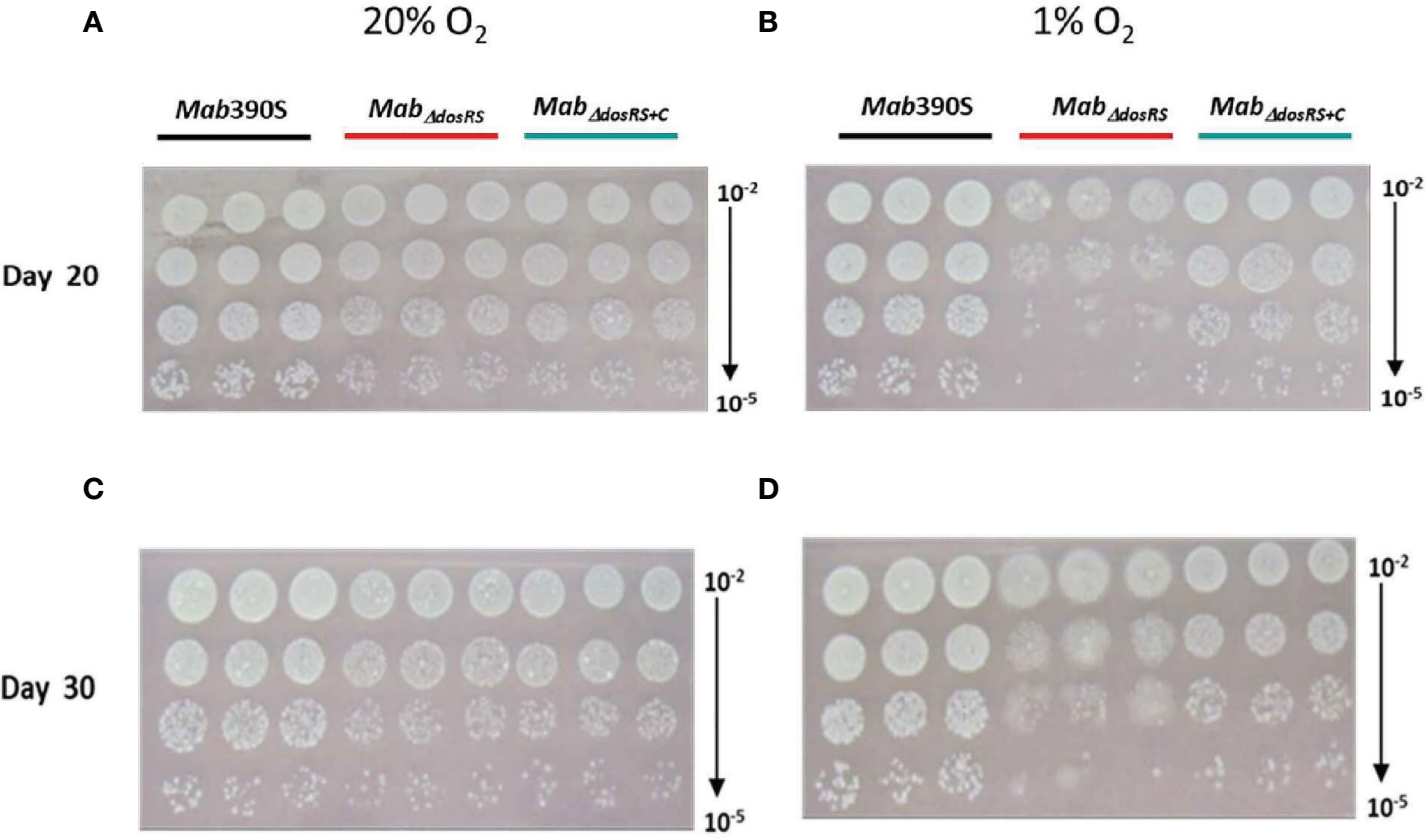
Hypoxia-induced morphological changes in *Mab_ΔdosRS_*. Cultures grown at 20% or 1% O_2_ were spot plated on Day 20 and Day 30 and incubated under normoxic conditions. **(A)** Day 20 at 20% O_2_ **(B)** Day 20 at 1% O_2_ **(C)** Day 30 at 20% O_2_ **(D)** Day 30 at 1% O_2_.

As detailed above, to verify the role of DosRS under *in vivo* relevant conditions, we compared the growth kinetics assays of *Mab* 390S, *Mab_ΔdosRS_*, and *Mab_ΔdosRS_*_+C_ in hypoxic (1% O_2,_ standing) versus aerobic (20% O_2_, shaking) conditions. Strains were monitored over a 30-day period using CFU/ml as the readout at day 5, 20, and 30 and grown in normoxic conditions after plating. Cultures grown in 20% O_2_ reached maximum growth on day 5 with no difference in growth between strains at any time point confirming fully aerated cultures are not dependent on DosRS for replication (**Figure 3A**). At 1% O_2_ maximum growth was achieved by day 20 in *Mab* 390S with a ~2-log decrease in *Mab_ΔdosRS_* and ~log decrease in *Mab_ΔdosRS_*_+C_ (**Figure 3B**) suggesting a functional DosRS is required for maximal growth and survival during hypoxic stress. By day 30 in 1% O_2_, *Mab* 390S displayed a slight decrease in CFU compared to day 20 however, this decline was also observed in fully aerated cultures indicating that hypoxic stress was not the cause (**Figures 3A, B**). These data support the conclusion that DosRS*_Mab_* is necessary for growth in hypoxic environments that mimic the physiologic environments of the CF lung, within macrophages and granulomas (Worlitzsch et al., 2002; Cunningham-Bussel et al., 2013; Hudock et al., 2017). Unexpectedly, a morphotype transition from smooth to rough occurred in *Mab_ΔdosRS_* after pro-longed exposure to 1% O_2_. On day 20 and 30 an observable change in morphology occurred only in the mutant strain resulting in a heterogenous population of smooth and rough colonies (**Figures 4A–D**), indicating a DosR-dependent inducible alteration in cell wall composition for *Mab_ΔdosRS_*. This is corroborated by the fact that in hypoxic liquid culture assays, *Mab_ΔdosRS_* alone adopted a biofilm-like pellicle layer that was resistant to disruption, whereas other strains maintained a homogeneous composition (data not shown).

**Figure 5:**
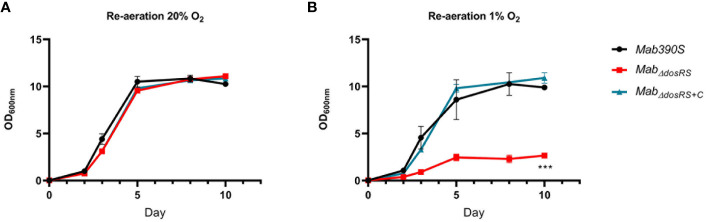
DosRS is required for resuscitation after hypoxia. OD_600nm_ was taken over a 10-day period of re-aerated cultures after 30 days of growth in either **(A)** 20% O_2_ or **(B)** 1% O_2._ *Mab390S* (black)*, Mab_ΔdosRS_* (red) and *Mab_ΔdosRS+C_* (blue). Data reflects 2 independent experiments performed in triplicate. *P*-values were calculated *via* one-way ANOVA using GraphPad, ***P-value <.001.

Prompted by the inability of *Mtb*_ΔdosR_ to resuscitate after re-aeration from hypoxia (Leistikow et al., 2010; Veatch and Kaushal, 2018), we investigated the ability of *Mab_ΔdosRS_* to resuscitate after 30 days in hypoxia. Day 30 cultures taken from 20% O_2_ and 1% O_2_ were diluted to an OD_600nm_ of 0.02 and grown in 20% O_2_ while shaking to evaluate the recovery of *Mab_ΔdosRS_* after prolonged exposure to hypoxia. OD_600nm_ was taken over a 10-day period (Day 0, 2, 3, 5, 8, and 10) to monitor growth kinetics after re-introduction of O_2_. All strains originating from aerobic conditions displayed similar growth curves after re-culturing and reached maximal OD by day 5 (**Figure 5A**). In contrast, after being subjected to hypoxia for 30 days, *Mab_ΔdosRS_* displayed reduced growth compared to *Mab* 390S and *Mab_ΔdosRS+C_* taken from 1% O_2_ (**Figure 5B**). Attenuated growth in 1% O_2_ and the inability to resuscitate after re-aeration for *Mab_ΔdosRS_* supports a critical role for DosRS*_Mab_* in mediating adaptation to changing oxygen levels encountered within the host during both dormancy and reactivation.

### Identification of a large and unique gene set regulated by DosRS*_Mab_*

**Figure 6:**
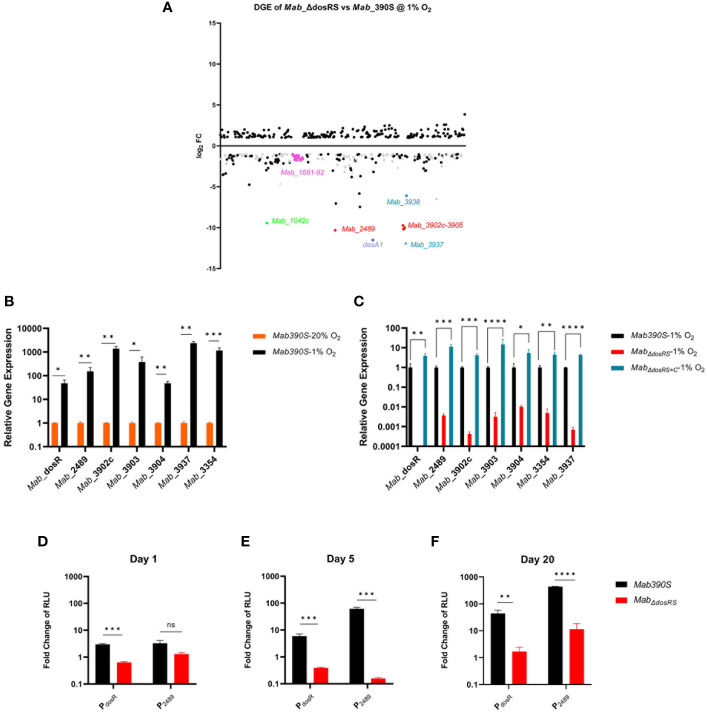
*Mab* DosR dependent DGE in 1% O_2_. **(A)** Scatter plot of RNAseq analysis of *Mab_ΔdosRS_* vs *Mab* 390S after 5 days in 1% O_2_. Figure shows genes with log_2_FC ≤ -1 and ≥ 1. Gray circles are conserved hypothetical genes and gray triangles are *Mab* genes with no ortholog in *Mtb.* Names of select genes of interest are labeled (neon green=*Mab*_*1042C*, pink=*Mab_1681-1698*, purple= *desA1*, turquoise (triangle denotes no known ortholog in *Mtb)* =*Mab_3937* & *3938*, red= predicted DosR regulated genes (RG). **(B, C)** qRT-PCR assessment of select DosR-dependent genes. Gene expression was calculated as relative quantitation *via* the 2^-ΔΔCt^ method using *sigA* as the reference gene. **(B)** Hypoxia induced gene expression. Transcript levels of select genes in *Mab* 390S 20% O_2_ (orange bars) and *Mab* 390S 1% O_2_ (black bars). **(C)** DosR dependent gene expression. Transcript levels of select genes in *Mab_ΔdosRS_* (red bars) and *Mab_ΔdosRS+C_* (blue bars) were compared to *Mab* 390S (black bars) in 1% O_2_ on Day 5. **(D-F)** Kinetics of DosR-dependent gene induction measured luciferase reporter assays. Promoter activity in 1% O_2_ was quantified by measuring luminescence compared to 20% O_2_ cultures and normalized to lux-hsp60 constitutive promoter as a reference signal, (1%O_2_(P*_dosR_* or P*_2489_*/P*_hsp60_*))/(20% O_2_(P*_dosR_* or P*_2489_*/P*_hsp60_*)) and expressed as fold change of relative light units (RLU) **(D)** 24hours, **(E)** Day 5, and **(F)** Day 20. *Mab* 390S (black bars) and *Mab_ΔdosRS_* (red bars). qRT-PCR and luciferase assay data are representative of 3 independent assays performed in triplicate. *P*-values were calculated using t-test **(B, D–F)** and one-way ANOVA **(C)**. *P*-values were calculated *via* GraphPad, *P-value <.05, ** *P*-value <.01, ***P-value <.001, ****P-value. Not significant is denoted as ns.

We next analyzed DGE between *Mab*390S and *Mab_ΔdosRS_* in hypoxia *via* RNAseq to experimentally identify DosRS regulated genes (**Figure 6A**). Cultures of *Mab* 390S and *Mab_ΔdosRS_* were grown at 1% O_2_ for 5 days at which point RNA was extracted from three independent experiments for analysis. In the absence of DosRS, 216 genes were expressed at lower levels relative to *Mab* 390S after exposure to 1% O_2_ (**Figure 6A**), of which 127 genes were also induced in *Mab* 390S by hypoxia (**Table S3**). This pattern is consistent with DosRS-dependent hypoxia induction, suggesting that *Mab* DosR may control a much larger regulon than previously predicted. In subsequent analyses, we defined putative DosRS-dependent hypoxia induced genes as those whose transcript levels were decreased by log_2_FC ≥ 1 in the *Mab_ΔdosRS_* in 1% O_2_ and were induced by log_2_FC ≥ 1 in *Mab* 390S 1% O_2_ (**Table S3**). The top 20 putative DosRS-dependent genes induced most highly by hypoxia (**Table S5**) include 4 of the genes previously predicted *in silico* to be members of the DosRS*_Mab_* regulon. Notably, two of the most highly upregulated genes, *Mab*_*3937* and *Mab*_*3354* (*desA1*), appear to be *Mab*-specific members of this regulon. *Mab*_*3937*, a hypothetical protein with no known ortholog, is predicted to be in an operon with *Mab*_*3938* and *Mab*_*3939*, encoding a clp protease subunit (ClpC2) with orthologous counterparts in *Mtb* and *Msmeg* which are essential genes. (Sassetti et al., 2003; Miranda-CasoLuengo et al., 2016; Kester et al., 2021). Both *desA1* (*Mab*_*3354*) and *desA2* (*Mab*_*1237*), desaturase enzymes with predicted roles in the biosynthesis of the mycolic acid component of mycobacterial cell walls, exhibited hypoxic DGE in the *dosRS* mutant (Yeruva et al., 2016; Bailo et al., 2022). Recently *desA2* but not *desA1* was predicted to be an essential gene in *Mab*, *Mtb* and *Msmeg* suggesting even slight downregulation could lead to detrimental alterations in the cell wall (Akusobi et al., 2022; Bailo et al., 2022). Although *Mtb* has orthologs of these genes (ClpC2, *desA1*, and *desA2)*, there is no evidence of regulation by DosR*_Mtb_*, illustrating the potential for conserved TCS to interact with conserved target genes in distinct ways. Additionally, no known DosR*_Mtb_*-regulated genes have been deemed essential, further highlighting the unique nature of DosRS*_Mab_*-mediated hypoxia response. Among the 127 hypoxia-induced putative DosR_Mab_-dependent genes is a large gene cluster (*Mab*_*1681-1698)* containing hypothetical proteins, daunorubicin resistance efflux pump subunit (*drrA)*, and a putative *mce* operon (**Table S5**). Whereas some putative *Mab* MCE gene clusters have clear orthologs in *Mtb* (e.g. MAB_4146c-4155c with Mce4, Rv3492c-3501c), the predicted *Mab* MCE proteins encoded by MAB_*1681*-*1698* do not directly correspond to a specific MCE loci in *Mtb.* Rather, they share low homology with components of different *Mtb* MCE complexes, necessitating further research to fully assess the role of this *Mab*-specific MCE during hypoxia.

Comparing the ~ 50 genes of the DosR*_Mtb_* regulon with the putative DosR*_Mab_* regulated genes identified in this study (**Supplemental Table 3**), we only discovered 6 shared orthologs including 4 conserved hypothetical proteins (CHPs) plus DosR (*Mab*_*3891c*) and DosS (*Mab*_*3890c)*. In addition to the transcriptional regulator *Mab*_*3891c*, we found nine other transcriptional regulators to be induced by hypoxia in a putative DosR-dependent manner (**Tables S3–5**) with none of their *Mtb* orthologs regulated by DosR*_Mtb_*. The transcriptional response of Mab to hypoxia is further differentiated from Mtb by the lack of regulation of any of the 7 putative triacylglycerol synthases (Tgs) by hypoxia or DosR, a characteristic of in vitro dormancy and hypoxia for Mtb (**Table S3**) (Voskuil et al., 2004; Deb et al., 2009). Hypoxic *Mtb* positively regulates *tgs1 via* DosR for synthesis of triacylglycerol (TAG) for energy storage and utilization. The absence of DosR*_Mab_* mediated induction of any Tgs enzymes under hypoxic stress points to a different mechanism for energy storage and utilization in *Mab* vs *Mtb* (Park et al., 2003; Daniel et al., 2004). The scope of the *Mab* DosR regulon precludes a comprehensive discussion of every downstream gene, many of which encode uncharacterized conserved hypothetical proteins. However, this RNAseq dataset is evidence for a species-specific regulon larger than predicted bioinformatically that likely contains novel mechanisms of hypoxia adaptation and pathogenesis.

Comparative transcriptomics also revealed upregulation of 200 genes in *Mab_ΔdosRS_* compared to *Mab* 390S (**Figure 6A** and **Table S3**), however the magnitude of induction was low ranging between log_2_FC of 1-3.6. Among the upregulated genes are 81 hypothetical proteins, 5 transcription factors, 30S and 50S ribosomal proteins. Included in the top 20 most highly induced genes in *Mab_ΔdosRS_* are *Mab_3521c* (nitrite reductase) and *Mab_3523c* (nitrite extrusion protein) which were observed to be highly downregulated in hypoxic *Mab* 390S (**Table S3**). These data suggests that DosRS may act as a repressor of a subset of genes in hypoxic conditions, a hypothesis that remains to be experimentally validated. The low-level induction of other genes in the knockout strain may also result from indirect effects of DosRS on mycobacterial physiology under hypoxic stress.

To validate RNAseq results, two of the putative *Mab* DosR-dependent genes most differentially expressed in the mutant strain (*Mab*_*3354* and *Mab*_*3937*) were assessed *via* qRT-PCR along with 5 of the predicted *Mab* DosR genes (**Figures 6B, C**). Similar to RNAseq studies, the effect of hypoxia on gene expression was assessed in *Mab* 390S (1% vs 20% O_2_) and the requirement for DosRS for DGE in response to hypoxia by comparing *Mab* 390S, *Mab_ΔdosRS_* and *Mab_ΔdosRS_*_+C_. All genes assessed *via* qRT-PCR were induced by ≥ 10-fold in 1% O_2_ compared to 20% O_2_ for *Mab* 390S with *Mab*_*3937*, *Mab*_*3902c*, and *Mab*_*3354* having the highest gene induction consistent with RNAseq results (**Figure 6B**). These same genes displayed loss of induction in *Mab_ΔdosRS_* by ≥ 10-fold similar to RNAseq results with restoration by *Mab_ΔdosRS+C_* in hypoxia (**Figure 6C**). The most dramatic change in gene expression in *Mab_ΔdosRS_* was *Mab*_*3902c* and *Mab*_*3937* with > 100-fold reduction compared to *Mab* 390S. Results from qRT-PCR corroborate RNAseq results and accentuate the magnitude of differential gene expression of predicted and newly discovered hypoxia-induced DosRS*_Mab_* regulated genes.

In addition to qRT-PCR, bacterial luciferase (Lux) reporter strains were used to evaluate the kinetics of hypoxia-dependent changes in gene expression across a broader time course. The integrating shuttle plasmid pMV306 luxG13 optimized for mycobacteria consists of the constitutive P_hsp60_ and P_G13_ promoters driving expression of *luxAB* and *luxCDE*, respectively (Andreu et al., 2010). Lux reporter constructs in which the constitutive P_hsp60_ promoter was replaced with promoters from two hypoxia inducible genes, DosR and *Mab*_2489 (P_DosR_ and P_2489_) were introduced into *Mab* 390S and *Mab_ΔdosRS_*. Reporter assays performed on Days 1, 5, 20 identified temporal changes in gene expression with both promoters for *Mab* 390S but not in *Mab_ΔdosRS_* (**Figures 6D–F**). The lux reporter under the control of P_dosR_ shows sustained induction over time, indicating that *dosR* is expressed throughout early and late stages of hypoxia, a trait not observed in *Mtb* (Rustad et al., 2008). Although the *Mab* 390S P_2489_-lux reporter displays modest induction of ~ 3-fold change on day 1, there is not a significant difference compared to *Mab_ΔdosRS_* P_2489_ (**Figure 6D**). However, on days 5 and 20 *Mab* 390S P_2489_-lux was highly induced compared to *Mab_ΔdosRS_* P_2489_-lux with fold changes of 61 and 433, respectively (**Figures 6E, F**). It should be noted that *Mab_ΔdosRS_* P_2489_-lux did exhibit low-level induction on Day 20, which may be attributable to the activity of other transcription factors. However, as noted previously, there was a significant difference compared to the expression of P_2489_ in wild-type *Mab* 390S. Lux reporters facilitated dynamic monitoring of DosRS activation by hypoxia and provide a valuable tool to explore DosR-mediated gene regulation *in vitro* and *in vivo* in response to various stresses (e.g. NO, CO, antibiotics) or host microenvironments (e.g intramacrophage, granuloma, airway mucus).

## Discussion

Infections caused by *Mab*, particularly within the CF population, are a major cause of concern due to the lack of efficacious antibiotics and the resulting inability to clear the infections from the airways. The poor correlation between *in vitro* drug susceptibility profiles and *in vivo* efficacy when treating *Mab* infections suggest that host-driven adaptations of *Mab* may contribute to treatment failures (Nessar et al., 2012). Host-derived cues encountered by *Mab* within the viscous mucus layer of CF airways, phagosomal compartments of macrophages, and during residence within granulomas may trigger upregulation of antimicrobial resistance mechanisms (Lyczak et al., 2002; Worlitzsch et al., 2002; Cunningham-Bussel et al., 2013; Hudock et al., 2017). Extrapolating from studies on *Mtb* (Gold and Nathan, 2017; Boldrin et al., 2020; Joshi et al., 2021)*, in vivo* stresses such as hypoxia may also promote the development of phenotypically drug-tolerant persisters. Thus, a better understanding of *Mab’s* physiological states and stress responses required for long-term persistence within the human host may lead to more effective treatment strategies.

Successful bacterial pathogens like *Mtb* and *Mab* employ extensive repertoires of transcription factors, including TCS, for coordinating gene expression to counteract host antimicrobial factors and immune pressure. The transcriptional regulatory networks and role of TCS of *Mtb* have been extensively characterized in multiple *in vitro* and *in vivo* models of infection (Bacon and Marsh, 2007; Rohde et al., 2012; Li et al., 2019; Stupar et al., 2022; Vilcheze et al., 2022). In contrast, few transcriptomic studies defining *Mab* stress responses, or the role of specific transcription factors have been reported (Miranda-CasoLuengo et al., 2016; Dubois et al., 2019). Given the well-documented relevance of hypoxia during *Mab-*host interactions (e.g. mucus of CF airway, macrophage phagosome, granuloma) (Lyczak et al., 2002; Worlitzsch et al., 2002; Cunningham-Bussel et al., 2013; Hudock et al., 2017), we sought to identify molecular mechanisms that enable *Mab* to adapt to these low-oxygen niches. In the better characterized pathogen *Mtb*, the master regulator of hypoxia adaptation is the atypical TCS DosRS/T, which regulates a ~50 gene regulon upon induction by hypoxia and NO stress (Park et al., 2003; Voskuil et al., 2003). *Mab* encodes orthologs of eleven of the twelve TCS found in *Mtb*, including DosRS (*dosT* homolog missing). However, as detailed in this study, the scope and content of *Mab* regulons controlled by these TCS may be less conserved. Prior to initiation of our study, only two reports had mentioned *Mab* DosRS: i) a bioinformatics study predicting a 6 gene regulon based on previously known *Mtb* DosR binding motifs (Gerasimova et al., 2011) and ii) transcriptomic study assessing the affect of NO exposure on the predicted genes (Miranda-CasoLuengo et al., 2016). These studies, however, did not directly demonstrate DosRS-mediated regulation of the predicted genes, define the transcriptional response of *Mab* to hypoxia nor the full extent of the DosR regulon, or identify a DosRS phenotype. To begin to address these knowledge gaps we developed a hypoxic model of 1% O_2_ to mimic physiologically relevant oxygen tensions *Mab* encounters *in vivo* (Worlitzsch et al., 2002; Cunningham-Bussel et al., 2013; Hudock et al., 2017) to assess transcriptomics and growth kinetics of *Mab* in the presence or absence of DosRS*_Mab_.*

Genome wide transcriptomics identified DosRS as the main TCS activated during hypoxia and analysis of a defined mutant lacking DosRS revealed a potentially larger regulon than previously predicted. We identified 216 genes to be downregulated in *Mab_ΔdosRS_* versus *Mab* 390S, 127 of which were upregulated in hypoxia. This gene set was deemed the putative hypoxia-induced DosR regulon-*Mab_3902c*, *Mab_3903* and Mab_*3904*), 2 novel genes displaying the highest DGE (MAB_3937 and *desA1*), 9 transcription factors, and 57 hypothetical genes among others. 22 of the 57 hypothetical genes are species-specific, further illustrating the unique nature of the regulons controlled by orthologous TCS. Not only is the *Mab* DosRS regulon likely larger than previously predicted, but it also appears to be notably larger than the well-studied *Mtb* DosR regulon. Surprisingly, the only orthologs in common between the *Mtb* DosR and *Mab* DosR regulons were the 6 genes originally predicted from the bioinformatics study and *Mab_1040*, an ortholog of the hypothetical protein *Rv3129* which is documented as an antigen in tuberculosis patients with latent infections (Park et al., 2003; Black et al., 2009; Lin et al., 2009). One of the hallmarks of *Mtb* hypoxia adaptations *in vivo* and *in vitro* is the marked upregulation of *tgs1* for energy storage and utilization (Garton et al., 2008; Deb et al., 2009). The induction of *Mab_3551c*, the primary TAG synthase gene in *Mab* (Viljoen et al., 2016), is not observed in our *Mab in vitro* hypoxia model, suggesting *Mab* mechanisms of hypoxia adaptation or cues for regulation of lipid storage are distinctive from *Mtb. *Studies are underway to discriminate between genes whose expression is altered directly by DosR (DosR binding to promoter) versus indirectly (promoter regulated by secondary TF).

During the course of our study, Belardinelli et al. also reported on characterization of the *Mab* DosR regulon as part of efforts to repurpose antimalarial drugs that inhibit *Mtb* DosR as therapeutics for *Mab* (Belardinelli et al., 2022). Their transcriptomic comparison of *Mab* ATCC 19977 and *Mab_ΔdosRS_* in microaerobic conditions identified 180 genes downregulated in a DosRS-dependent manner. Of these 180 genes, only 45 overlapped with our list of 216 genes downregulated in the *dosRS* mutant at 1% O_2._ Both studies included the 6 previously predicted genes and the 2 genes most highly differentially expressed on our list (*Mab*_*3937*, *desA1*) plus 37 other genes. Of the 45 genes in common between these 2 studies, Belardinelli et al., reported 38 DosR binding motifs supporting the assertion that *Mab* DosR regulon is larger than previously predicted. Discrepant findings between this report and our study could be attributable to differences in strains (ATCC 19977 vs 390S), hypoxic models (20% O_2_ standing vs 1% O_2_ standing), time points (24hr vs 5 days), or media (Dubos-Tween albumin vs 7H9-OADC+.05% tyloxapol). Regardless, both clearly highlight the broad scope and unique nature of the DosRS*_Mab_* regulon and provide a framework for future studies to fully elucidate the role of this important two-component system.

The importance of DosR-regulated genes for hypoxia adaptation was evident from growth deficits seen on day 20 and 30 and impaired resuscitation after reaeration in *Mab_ΔdosRS_* compared to *Mab* 390S (**Figure 3**). *Mab_ΔdosRS* differentially expressed genes in hypoxia contain 7 genes predicted to be essential in a recent TnSeq study under aerobic conditions (Akusobi et al., 2022), possibly accounting for these phenotypes. Included in this list is *desA2*, a desaturase enzyme that is responsible for mycolic acid biosynthesis and is essential for growth in the RGM *Msmeg* (Bailo et al., 2022). Strong induction of *desA1* in hypoxia suggests that it, along with *desA2*, may play role in cell wall modification in response to this stress. It is worth noting that, despite exclusion from the list of *Mab* predicted essential genes, the orthologous desaturase in *Msmeg* was deemed essential (Singh et al., 2016). Additionally, the MCE operon *Mab*_1693-*Mab*_1698 was differentially expressed in the mutant strain and may contribute to decreased importation of mycolic acids further disrupting cell wall integrity. The 6 other predicted essential genes possibly contributing to the *Mab_ΔdosRS_* growth phenotype are 2 conserved hypothetical proteins (*Mab_3268c*-*Mab_3269c*), a DNA helicase (*Mab_3511c*), a protoporphyrinogen oxidase, a prephenate dehydratase (*Mab_0132*), and phosphoribosylformylglycinamidine synthase (*Mab_0698*).

In addition to growth/survival deficits and the inability to resuscitate, we also observed DosRS-dependent hypoxia-induced morphological changes. After 20 days in hypoxia, *Mab_ΔdosRS_* displayed heterogeneous morphology consisting of smooth and rough colonies. This phenotype was not present in strains expressing DosRS or in fully aerated cultures, evidence that this TCS mediates dramatic remodeling of the cell wall in response hypoxia. The smooth and rough morphotypes of *Mab*, reflective of different compositions of the outer cell wall, have been shown to impact interactions with macrophages, immune stimulation and inflammation, antibiotic susceptibility, and virulence (Jonsson et al., 2007; Catherinot et al., 2009; Ruger et al., 2014; Roux et al., 2016; Li et al., 2020). Rough strains are able to trigger apoptosis of macrophages and grow extracellularly as aggregates known as cords, and are associated with worse clinical outcomes (Li et al., 2020) Mechanisms involved in smooth to rough transitions have not been fully elucidated. However, genomic comparisons between the two morphotypes revealed SNPs and indels in the *gpl* locus and in *mmpl4b* and *mps1* genes (Pawlik et al., 2013). In addition to total or partial loss of GPL due to mutations affecting its biosynthesis or transport, recent studies including identification of GPL+ rough clinical isolates suggest other mechanisms may also govern S➔R morphotype switching (Gutierrez et al., 2021). For example, Daher et al. reported that glycosylation patterns of GPL can alter *Mab* surface properties (Daher et al., 2022). An inducible transition from smooth to rough was also observed following exposure to subinhibitory doses of aminoglycoside antibiotics providing evidence for transcriptional modulation of morphotype in response to stress (Tsai et al., 2015). Belardinelli et al. reported no differences in GPL content between *Mab* ATCC 19977 and isogenic *ΔdosRS* mutant after microaerobic culture for 24 hours (Belardinelli et al., 2022). Our observation of a switch to rough morphotype in a *ΔdosRS* mutant after extended culture at 1% O_2_ may reflect either adaptive cell wall remodeling triggered by lower O_2_ levels or longer duration of stress in our model. Alternatively, rather than affecting GPL levels per se, inactivation of the DosRS regulon may impact GPL modifications or biosynthesis of unique cell wall constituents. Intriguingly, Pawlik et al. reported that expression of *dosR* was elevated in an R versus S strain (Pawlik et al., 2013). This seems to contrast with our data suggesting that DosRS positively regulates GPLs, or at least the smooth phenotype (e.g. loss of DosRS➔rough phenotype in hypoxia). Whether this is a direct correlation or stress induced side-effect stemming from the loss of GPL remains to be determined. It is clear that much remains to be learned regarding how *Mab* regulates the composition of its complex cell wall during infection and the roles of TCS like DosRS in host-pathogen interactions.

In addition to determination of the DosRS-dependent component of *Mab* hypoxia adaptation, this is the first transcriptomics study designed to identify genome-wide changes in *Mab* gene expression in a defined, physiologically relevant model of hypoxia. RNAseq analysis of wild-type *Mab*390S in 1% O_2_ versus 20% O_2_ identified an additional 1,063 DosRS-independent hypoxia-induced genes with putative roles in *Mab* adaptation in hypoxia. Differential gene expression of such a large group of genes in hypoxia outside of the DosR regulon points to a sophisticated mechanism of regulation for adaptation beyond TCS. This gene set included 80 TF, lipid metabolism and transport, energetics, secondary metabolism, cell wall synthesis plus induction of 540 hypothetical proteins, (**Table S3**). Our data highlights the necessity of adaptation to hypoxia *via* a large repertoire of genes including but not limited to the TCS DosRS. Further investigation of unique *Mab* DosR regulated genes and species-specific *Mab* genes employed for hypoxic adaptation will provide beneficial insights into *Mab* pathogenesis.

## Data availability statement

The datasets presented in this study are deposited in the NCBI database, accession number PRJNA932814 (https://www.ncbi.nlm.nih.gov/bioproject/?term=PRJNA932814).

## Author contributions

BS and KR: Conception and design of experiments. BS and BT: Analysis and interpretation of data for RNAseq experiments. BS and KR: Preparation and revision of manuscript. KR and LS: Approval of final manuscript. All authors contributed to the article and approved the submitted version.
