# Supplementary figures and images for: Mycobacterium abscessus DosRS two-component system controls a species-specific regulon required for adaptation to hypoxia
Source: Front Cell Infect Microbiol. 2023 Mar 9;13:1144210. doi: 10.3389/fcimb.2023.1144210 (PMC10034137; doi:10.3389/fcimb.2023.1144210)

a)

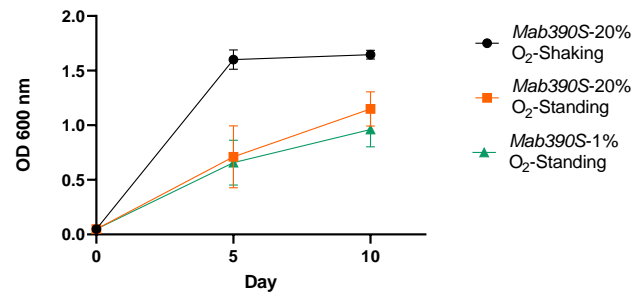

Supplement: Supplementary file 1 [file DataSheet_1.pdf]
